# Antibiogram of Bacteria Isolated from Bloodstream Infection-Suspected Patients at the University of Gondar Comprehensive Specialized Hospital in Northwest Ethiopia: A Retrospective Study

**DOI:** 10.1155/2024/7624416

**Published:** 2024-07-08

**Authors:** Minichil Worku, Tigist Molla, Desie Kasew, Muluneh Assefa, Alene Geteneh, Melak Aynalem, Mucheye Gizachew, Sirak Biset

**Affiliations:** ^1^ University of Gondar Comprehensive Specialized Hospital, Gondar, Ethiopia; ^2^ Department of Medical Microbiology School of Biomedical and Laboratory Sciences College of Medicine and Health Sciences University of Gondar, Gondar, Ethiopia; ^3^ Department of Medical Laboratory Science College of Health Sciences Woldia University, Woldia, Ethiopia; ^4^ Department of Hematology and Immunohematology School of Biomedical and Laboratory Sciences College of Medicine and Health Science University of Gondar, Gondar, Ethiopia

## Abstract

**Background:**

Bacterial bloodstream infections (BSIs) are the leading cause of sepsis-related morbidity and mortality worldwide. The emergence and spread of antimicrobial resistance (AMR) in bacteria is also a growing global concern. As a result, data on bacterial profile and their antibiogram are essential for strategies to contain drug resistance, improve the quality of patient care, and strengthen health systems.

**Methods:**

Retrospective data from bacteriological results of blood samples of BSI-suspected patients from 2018 to 2021 were collected using a data collection sheet. Standard bacteriological techniques were followed during sample collection, culture preparation, bacterial identification, and antibiotic susceptibility testing (AST). We used Epi Info version 7 to enter and clean the data and then exported it to SPSS version 26 for analysis. Logistic regression models were used to measure the association between variables. A *p* value <0.05 with a 95% confidence interval was considered as statistically significant.

**Result:**

Of the total 2,795 blood culture records, 455 (16.3%) were culture positive for bacteria, with *Klebsiella pneumoniae* (26%) and *Staphylococcus aureus* (24.6%) being the leading isolates. The isolates were highly resistant to common antibiotics, with more than 80% of them being resistant to ceftriaxone and penicillin. Moreover, about 43% of isolates were multidrug resistant (MDR), with *Klebsiella pneumoniae* (65.5%), *Acinetobacter* species (56.7%), and *Citrobacter* species (53.8%) being the most common MDR isolates. Age and diagnosis year were significantly associated with the presence of bacterial BSIs (*p* value <0.05).

**Conclusion:**

Bacterial BSI and AMR were growing concerns in the study area. Bacteremia was more common in children under the age of five, and it decreased as the patient's age increased. The alarming rate of AMR, such as MDR blood isolates, calls for periodic and continuous monitoring of antibiotic usage in the study area.

## 1. Introduction

Sepsis, a life-threatening organ dysfunction caused by a dysregulated host response to infections, is the major global health threat with a high morbidity and mortality rate, particularly in sub-Saharan Africa (SSA) [1]. In 2017, there were about 49 million sepsis-related incident cases, 41% of which occurred in children under the age of five, and 11 million sepsis-related deaths, accounting for 20% of all annual deaths worldwide [2]. Infections, particularly bacterial bloodstream infections (BSIs), have remained the leading cause of sepsis and sepsis-related mortality worldwide across all ages. The presence of viable microorganisms in the bloodstream results in an inflammatory response characterized by changes in clinical, laboratory, and hemodynamic parameters [3–5]. Although the presence of viable bacteria in the bloodstream (bacteremia) is the commonly reported BSI worldwide, other organisms such as fungi, viruses, and parasites can also be involved [3].

In recent years, treating infections such as bacteremia has become a nightmare for the entire world due to the increasing resistance of the causative agents to most of the essential antimicrobials for human medicine [6]. Indeed, one of the biggest threats to public health in the 21^st^ century is the emergence of antimicrobial resistance (AMR) in bacteria, which occurs when changes in bacteria lead to a reduction in the effectiveness of the drugs used to treat infections [7]. Infections due to AMR bacteria result in death, longer duration of hospitalization, and pose a significant economic burden on national healthcare systems [8]. For instance, in 2019, around 4.95 million deaths were associated with bacterial AMR, with the highest rate registered in low-resource settings, particularly SSA [9]. Globally, the leading pathogens causing bacteremia are *Escherichia coli* (*E. coli*), *Staphylococcus aureus* (*S. aureus*), *Klebsiella pneumoniae* (*K. pneumoniae*), *Pseudomonas aeruginosa* (*P. aeruginosa*), *Enterococcus* species, *Acinetobacter baumannii* (*A. baumannii*), Coagulase-negative *staphylococcus* (CoNS), typhoidal and nontyphoidal *Salmonella* species, and others [10–13]. At least five of these bacteria are reported as the leading pathogens responsible for AMR-related deaths in 2019 [9].

The widespread and continuous emergence of AMR in bacteria causing BSIs coupled with the global spread of multidrug-resistant (MDR) strains is a major concern worldwide, particularly in low- and middle-income countries (LMICs) where testing coverage is low and healthcare systems are weak, which impacts AMR interventions [11, 13]. Given the global rise in BSIs and the evolving drug-resistance profiles of the organisms involved, data on pathogen profiles and their AMR patterns are essential for strategies to contain drug resistance, improve the quality of patient care, and strengthen health systems [11]. Aside from the potential consequences of BSIs, delays in performing and receiving culture results, as well as the lack of susceptibility patterns for local isolates, lead to the frequent use of empirical therapy, which in turn contributes to the emergence of AMR, the consumption of expensive agents, and issues related to drug toxicity [13]. As a result, identifying changes in pathogen distribution and AMR rates can help update diagnostic approaches, therapeutic strategies, and infection control and prevention measures in healthcare settings.

## 2. Materials and Methods

### 2.1. Study Setting, Design, and Period

A retrospective cross-sectional study was conducted at the University of Gondar Comprehensive Specialized Hospital (UoG-CSH) in northwest Ethiopia from May to June 2022. The study involved the collection of data from patient culture records in the bacteriology laboratory of the UoG-CSH, which is a teaching and referral hospital located in Gondar City, 750 km northwest of Addis Ababa, Ethiopia. This hospital is one of the largest medical facilities in the country, with over 1,200 beds. The hospital serves a population of more than 7 million people from Gondar and its surrounding areas. One of the notable features of the UoG-CSH is its accredited bacteriology laboratory, which plays a crucial role in the management of infectious diseases [14].

### 2.2. Study Population

We included all patients who were suspected for BSIs and had recorded blood culture results registered on the bacteriology culture registration books from January 2018 to December 2021. Records with incomplete patient and laboratory data were excluded from the study.

### 2.3. Data Collection

We conducted a retrospective review of four-year (January 2018 to December 2021) laboratory records of all blood cultures from patients suspected of having BSIs from all departments and units of the UoG-CSH. We used a data abstraction form to collect patients' sociodemographic and laboratory data (age, gender, diagnosis year, blood culture results, the isolated bacteria, and antimicrobial susceptibility testing (AST)) results from the laboratory record books.

### 2.4. Laboratory Methods

The UoG-CSH bacteriology laboratory used standard laboratory procedures to process blood culture and antimicrobial testing. Blood samples (10 mL for adults, 3 mL for children, and 1 mL for neonates) were collected by experienced healthcare workers from patients suspected of having BSIs using culture bottles with sterile tryptic soy broth (Oxoid Ltd., Basingstoke, UK). The culture bottles were then incubated at 37°C for 7 days and checked daily for signs of bacterial growth. Samples from culture bottles with signs of growth were then inoculated onto MacConkey agar, Blood agar, and Chocolate agar (BIO MARK Laboratories, India) and incubated for 24 hours. [15]. Growth from these media was characterized, and species were identified by a series of biochemical tests [16]. Once the species were identified, AST was carried out using Kirby–Bauer disc-diffusion technique [17] on Muller–Hinton agar (MHA) (Oxoid Ltd., UK) following the Clinical Laboratory Standards Institute (CLSI) guidelines (2017–2020). The bacterial suspension was standardized using 0.5 McFarland standard and inoculated on MHA (Oxoid Ltd., UK). The antibiotic discs were dispensed after drying the plate for 3–5 min and incubated at 37°C for 24 hours. Antibiotic discs such as penicillin (10 units), ampicillin/amoxicillin (20/10 *μ*g), ceftriaxone (30 *μ*g), cefuroxime (30 *μ*g), cefoxitin (30 *μ*g), meropenem (10 *μ*g), amikacin (30 *μ*g), clindamycin (2 *μ*g), tetracycline (30 *μ*g), gentamicin (10 *μ*g), vancomycin (30 *μ*g), chloramphenicol (30 *μ*g), erythromycin (15 *μ*g), ciprofloxacin (5 *μ*g), piperacillin-tazobactam (100/10 *μ*g), cefotaxime (30 *μ*g), ceftazidime (30 *μ*g), doxycycline (30 *μ*g), and trimethoprim-sulfamethoxazole (1.25/23.75 *μ*g) (HI Media Laboratories, India) were used to test the susceptibility of bacterial isolates.

### 2.5. Quality Control

The UoG-CSH bacteriology laboratory followed standard operating procedures during sample collection, transportation, and processing. The culture media were regularly checked for sterility by incubating five percent of the prepared media overnight and examining the presence of growth. Performances of the media were also checked by inoculating control strains before culture and sensitivity tests were performed. Selection of discs was based on local availability and following the CLSI guideline [18]. The reference strains such as *S. aureus* (ATCC 25923), *E. coli* (ATCC 25922), and *P. aeruginosa* (ATCC 27853) were used for quality control. The UoG-CSH bacteriology laboratory obtained all reference strains from the Amhara Public Health Institute (APHI), Bahir Dar, Ethiopia.

### 2.6. Data Analysis and Interpretation

Data were entered, cleaned, coded, and analyzed using SPSS version 26 software. Descriptive analysis was used to describe and calculate frequencies and percentages of variables. The strength of association between dependent and independent variables was assessed using the binary logistic regression model. Variables with a *p* value <0.20 during bivariate analysis were further checked in multivariate analysis to determine any associations, which were expressed by an adjusted odds ratio at a 95% confidence interval. Finally, results were presented in texts, tables, and graphs.

## 3. Results

### 3.1. Sociodemographic Characteristics

A total of 2,795 BSI-suspected patients, with registered data at the UoG-CSH bacteriology laboratory, were included in this study. The male-to-female ratio was 1.42 : 1, with 1640 (58.7%) being male. The mean age of the study subjects was 14.84 years (±19.7 SD), with 1487 (53.2%) being under the age of five, including 580 (20.8%) neonates and 535 (19.1%) infants. About 808 (28.9%) of patients were from the pediatric emergency department, 572 (20.5%) from the neonatal ward, and 512 (18.3%) from the pediatric ward. The prevalence of bacterial BSI was 455/2795 (16.3% (95% CI = 14.9–17.6)), with highest prevalence recorded in the pediatric emergency (163/808, 20.2%) and neonatal wards (134/572, 23.4%). The burden of bacterial infection increased from 69/571 (12.5%) in 2018 to 166/875 (19.0%) in 2021 (Table 1).

Age and diagnosis year were independently associated with the presence of bacterial BSI (*p* < 0.05). The odds of neonates, children aged one to five years, and infants having bacterial culture positive results were 3.4 (95% CI = 1.631–7.118, *p*=0.001), 3.17 (95% CI = 2.063–4.877, *p* < 0.001), and 3.03 (95% CI = 1.990–4.617, *p* < 0.001) times higher, respectively, than adults aged 19 to 45 years. Regarding the year of diagnosis, the odds of bacterial culture positive results in 2021 and 2020 were 1.7 (95% CI = 1.242–2.321, *p*=0.001) and 1.63 (95% CI = 1.144–2.326, *p*=0.007) times higher, respectively, than in 2018 (Table 2).

### 3.2. Distribution of Bacterial Isolates

**Figure 1:**
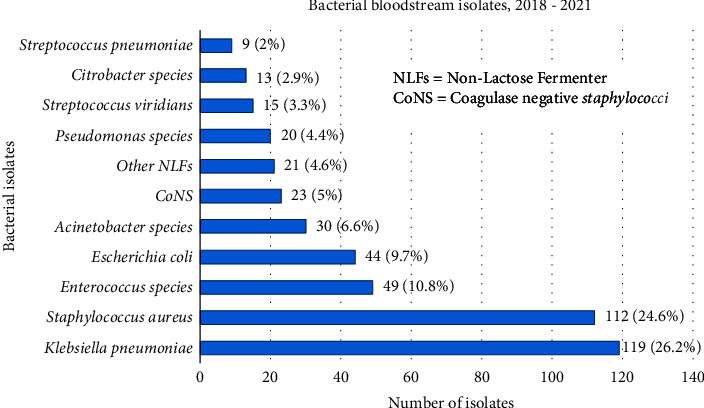
Distribution of bacterial isolates from blood culture of bacteremia-suspected patients at the UoG-CSH from January 2018 to December 2021, northwest Ethiopia.

The predominant isolates were *K. pneumoniae* (119/455, 26.15%), followed by *S. aureus* (112/455, 24.6%), *Enterococcus* spp. (49/455, 10.77%), and *E. coli* (44/455, 9.67%) (Figure 1).

The majority of the isolates, 247/455 (54.3%), were GNB, with the remaining 208/455 (45.7%) being GPB. The most common GPB were *S. aureus* (24.62%), *Enterococcus* species (10.7%), and CoNS (5.05%). On the other hand, *K. pneumoniae* (26.15%) was the most frequently isolated GNB, followed by *E. coli* (9.67%) and *Acinetobacter* species (6.6%). GPB isolates were most common in children aged one to five years (11.83%) and infants (11.78%), while GNB were most common in neonates (16.38%), children aged one to five years (10.5%), and infants (9.53%) (Table 3).

### 3.3. Antimicrobial Susceptibility Profile

The overall rate of resistance in GPB was 52.95%, ranged from 11.2% to 85.9%. The isolates were sensitive to chloramphenicol (84.3%), trimethoprim-sulfamethoxazole (80.0%), doxycycline (78.8%), ciprofloxacin (68.3%), amikacin (67.6%), and clindamycin (63.6%). However, they were highly resistant to penicillin (85.9%), piperacillin/tazobactam (83.3%), and tetracycline (75.9%). *Streptococcus viridans (S. viridans)* had the highest resistance rate (58.8%), followed by *Enterococcus* spp. (53.1%), *S. aureus* (52.9%), and CoNS (52.4%) (Table 4).

The overall rate of resistance in GNB was 63.6%, with a range of 24.7% to 84.5%. The isolates were sensitive to trimethoprim-sulfamethoxazole (75.3%), chloramphenicol (70.0%), and clindamycin (65.3%). However, they were highly resistant to ceftriaxone (84.5%), oxacillin (84.3%), ampicillin/amoxicillin (81.6%), and piperacillin/tazobactam (81.2%). *K. pneumoniae* showed a high level of overall resistance (70.4%) to the tested antimicrobials, followed by other nonlactose fermenter (NLF) rods (68.6%) and *Acinetobacter* spp. (67.3%) (Table 5).

### 3.4. Multidrug-Resistance Patterns

The overall prevalence of MDR was 43.5%. Gram-negative isolates (140, 56.7%) showed a higher MDR rate than Gram-positive isolates (58, 27.9%). The range of MDR rate among isolates was between 20.0 and 65.5%, with 65.5%, 56.7%, and 53.8% of *K. pneumoniae*, *Acinetobacter* spp., and *Citrobacter* spp. isolates were MDR, respectively (Table 6).

## 4. Discussion

Bloodstream infections, mainly caused by MDR bacteria, are life-threatening events that require immediate interventions [19, 20]. Thus, this study identified common bacteria and their AST pattern from BSI-suspected patients at the UoG-CSH. We found that bacteremia was a common healthcare problem in the study area, with under-five children being the most affected. The prevalence of bacteremia increased as the year progressed, with *K. pneumoniae* and *S. aureus* being the leading causative agents. Furthermore, more than 89% of the bacterial isolates were resistant to at least one antibiotic agent, including 92% of Gram-positive and 86.5% of Gram-negative isolates. We also found that about 43.5% of the bacterial isolates were MDR, with 56.7% of Gram-negative and 27.9% of Gram-positive isolates being MDR. These isolates were highly resistant to beta-lactam antibiotics such as penicillin and cephalosporins.

The overall prevalence of BSI in this study was 16.3% (95% CI = 14.9–17.6), which is comparable to reports in India (16.1%) [21] and Pakistan (16.6%) [22]. In contrast, it is higher than reports in Ethiopia [23, 24] and elsewhere [20, 25–29]. However, previous retrospective studies from the same study area, including the years 2001 to 2005 [30], 2006–2012 [31], and 2012 to 2018 [14], reported a higher prevalence of BSI. Other studies in other parts of Ethiopia (Mekelle, 2015, and Addis Ababa, 2015-2016) [32, 33] and elsewhere [34, 35] also revealed a higher prevalence of culture-confirmed bacteremia. The possible explanation for the variations of BSI prevalence across studies might be owing to differences in the number of blood culture samples included, study population, methodology, study area, and infection prevention and control practices.

We found a relatively higher prevalence of bacterial isolates in neonates (23.6%), followed by under-five children (22.3%) and infants (21.3%), with a higher prevalence of *S. aureus* and *K. pneumoniae*. This finding was in line with the study in Maldives, where neonates comprised a high proportion of BSI in comparison to infants and children [5]. Similarly, a study in Bangladesh depicted that *K. pneumoniae* (75.96%) was the predominant isolate among neonates with sepsis [36]. Similar to the current study, *K. pneumoniae* (40.4%) and *S. aureus* (17.0%) were identified as the most common bacterial pathogens of BSI in children in Tanzania [37]. This is an indication that the epidemiology of the previously leading bacterial etiologies of BSI has been changing possibly due to the implementation of vaccines and invasive treatment modalities [38, 39].

We also found that bacterial BSI was higher in the neonatal and pediatric emergency ward, accounting for 23.4% and 20.2%, respectively. This finding is comparable with previous reports (29.4% and 19.7%) from the same study area [40, 41]. However, it is higher than a report in Cameroon [42] and lower than reported in Rwanda [28]. The enrollment of large number of under-five children in the current study may contribute to the discrepancies seen between studies, as children are more likely to expose to microorganisms as a result of contaminated food, soil, and other materials, as well as a lack of personal hygiene [43]. Many studies showed that BSI is one of the most frequently observed infections in neonatal and pediatric wards. The increased risk of sepsis due to bacteria is related to the persistent immune dysfunction [44]. For example, in Europe, about 44.6% of healthcare-associated infections (HAIs) in children were reported as BSI, with a high prevalence in neonates and infants in their first 11 months of life [45]. Furthermore, infections caused by AMR bacterial pathogens are increasing in neonatal settings and infants admitted to the neonatal ward, particularly in those born preterm, with immature immunity, prolonged hospitalization, and frequent use of invasive medical devices [46].

The results of our study showed that GNB were responsible for the majority of BSIs (54.3%), with *K. pneumoniae* (26.15%) being the most common cause, followed by *S. aureus* (24.62%), *Enterococcus* species (10.7%), and *E. coli* (9.67%). Comparable to this finding, studies in Rwanda [28], India [34, 47], and Ethiopia [41] reported *K. pneumoniae*, *S. aureus*, and *E. coli* as leading causes of BSIs. Infection prevention and control practices, geographic variation, medical environment, socioeconomic status, population, and prior use of inappropriate broad-spectrum antimicrobials may affect the distribution of bacterial pathogens [48, 49]. The ability of *K. pneumoniae* to adapt the hospital environment, evade immune system, and resist antibiotics has led to an increase in its infection rate over the past few years [50]. In *K. pneumoniae*, a variety of antibiotic resistance mechanisms can be involved, including changes in cell permeability, target gene mutation, plasmid-mediated resistance, and production of enzymes such as betalactamases, carbapenemase, and aminoglycoside-modifying enzymes [51, 52]. Additionally, the higher prevalence of *K. pneumoniae* and *S. aureus* in this study is possibly due to their predominance in the study area [53].

Gram-positive bacterial isolates in this study were highly resistant to penicillin (85.9%), piperacillin/tazobactam (83.3%), tetracycline (75.9%), ampicillin/amoxicillin (66.7%), and erythromycin (66.7%). Comparable to this finding, a prospective study in Gondar reported a high resistance pattern of penicillin, ampicillin, and erythromycin in GPB [40]. This could be related to the fact that these pathogens are known to have vast genetic repertoire to adapt and develop AMR, which indeed requires the global efforts in recognizing the emerging AMR mechanisms as to optimize the use of antibiotics and create strategies to circumvent this challenge [54]. Gram-negative bacteria were also highly resistant to ceftriaxone (84.5%), oxacillin (84.3%), ampicillin/amoxicillin (81.6%), piperacillin/tazobactam (81.2%), gentamicin (76.9%), cefuroxime (76.7%), tetracycline (73.6%), erythromycin (66.7%), amikacin (62.5%), and ciprofloxacin (60.0%). Singh et al. [26] also reported that GNB and the members of *Enterobacteriaceae* showed more resistance to ampicillin (91.7%), ceftriaxone (88.5%), and gentamicin (60.9%).

The overall prevalence of MDR in this study was 43.5%, which is comparable to previous reports in Ethiopia [24, 32, 55]. Among the total MDR isolates, 56.7% were GNB and 27.9% were GPB. This could be due to the increased presence of AMR determinants such as extended-spectrum beta lactamase (ESBL) and carbapenemase in GNB isolates in hospital settings [56, 57]. Furthermore, MDR prevalence was higher in *K. pneumoniae* (65.5%), followed by *Acinetobacter* spp. (56.7%) and *Citrobacter* spp. (53.8%). The increasing trend of MDR may be due to the overuse and misuse of antibiotics, high load of infectious diseases, poor infection prevention and control practices, poor-quality antimicrobials, inadequate knowledge of AMR, misdiagnosis of etiologic agent, lack of advanced laboratories for AST, and poor hygiene and sanitation, especially in children [58–60].

### 4.1. Limitation of the Study

Although this study reports the main bacteria involved in BSIs and their antibiotic susceptibility patterns, which is essential to rationalize the empiric antimicrobial therapy at the study area, we recognize that it has limitations. The major limitation was attributable to the use of secondary data; due to the limited patient details recorded on the laboratory logbook, we could not access the full range of sociodemographic, behavioral, environmental, and clinical factors for bacterial BSI, which could affect the possible association that the factors may have with the presence of BSIs. The availability of antibiotic disks for antibiotic susceptibility testing was not consistent over the four-year period, which may affect the analysis of antimicrobial susceptibility pattern of isolates.

## 5. Conclusion and Recommendation

Bacterial BSI is a growing concern in the study area, with a considerable increase in prevalence as the year progressed. Bacteremia was more common in children under the age of five, particularly among neonates and infants, and reduced as the patient's age increased. *Klebsiella pneumoniae* and *S. aureus* were the leading causes of bacteremia, followed by *Enterococcus* spp. and *E. coli*. There was a high burden of AMR in the study area, with more than 80% of bacteremia-causing isolates becoming resistant to ceftriaxone and penicillin. Furthermore, more than half of Gram-negative isolates were MDR, with *K. pneumoniae*, *Acinetobacter* spp., and *Citrobacter* spp. being the leading MDR isolates. Therefore, routine bacterial identification and AST are recommended to detect the emergence and spread of AMR bacteria as early as possible.

## Figures and Tables

**Table 1:** Sociodemographic characteristics of patients at the UoG-CSH.

Variables		Frequency (%)	Bacterial infection	
			Positive (%)	Negative (%)
Sex	Male	1640 (58.7)	273 (16.6)	1367 (83.4)
	Female	1155 (41.3)	182 (15.8)	973 (84.2)

Age in years	<1 month	580 (20.8)	137 (23.6)	443 (76.4)
	1 month– <1 year	535 (19.1)	114 (21.3)	421 (78.7)
	1–<5 years	372 (13.3)	83 (22.3)	289 (77.7)
	5–12 years	281 (10.1)	38 (13.5)	243 (86.5)
	13–18 years	150 (5.4)	14 (9.3)	136 (90.7)
	19–45 years	635 (22.7)	51 (8.0)	584 (92.0)
	>45 years	242 (8.7)	18 (7.4)	224 (92.6)

Ward types	Pediatric emergency	808 (28.9)	163 (20.2)	645 (79.8)
	Neonatal ward	572 (20.5)	134 (23.4)	438 (76.6)
	Pediatric ward	512 (18.3)	53 (10.4)	459 (89.6)
	Adult ward	370 (13.2)	48 (13.0)	322 (87.0)
	Adult emergency	246 (8.8)	22 (8.9)	224 (91.1)
	Surgical and recovery	181 (6.5)	19 (10.5)	162 (89.5)
	Gynecology and obstetrics	106 (3.8)	16 (15.1)	90 (84.9)

Year of diagnosis	2018	551 (19.7)	69 (12.5)	482 (87.5)
	2019	872 (31.2)	133 (15.3)	739 (84.7)
	2020	497 (17.8)	87 (17.5)	410 (82.5)
	2021	875 (31.3)	166 (19.0)	709 (81.0)

Total (%)		2795 (100)	455 (16.3)	2340 (83.7)

**Table 2:** Factors associated with bacteremia at the UoG-CSH.

Variables		Bacteremia		COR (95% CI)	*p* value	AOR (95% CI)	*p* value
		Yes	No				
Gender	Male	273	1367	1.068 (0.870, 1.310)	0.531	—	—
	Female	182	973	1			

Age	<30 days	137	443	3.541 (2.510, 4.997)	<0.001^*∗*^	3.407 (1.631, 7.118)	0.001^*∗*^
	30 days–<1 year	114	421	3.101 (2.178, 4.414)	<0.001^*∗*^	3.031 (1.990, 4.617)	<0.001^*∗*^
	1–5 years	83	289	3.289 (2.258, 4.790)	<0.001^*∗*^	3.172 (2.063, 4.877)	<0.001^*∗*^
	5–12 years	38	243	1.791 (1.147, 2.797)	0.010^*∗*^	1.587 (0.963, 2.615)	0.070
	13–18 years	14	136	1.179 (0.634, 2.192)	0.603	0.992 (0.520, 1.890)	0.980
	19–45 years	51	584	1		1	
	>45 years	18	224	0.920 (0.526, 1.609)	0.771	0.875 (0.498, 1.537)	0.643

Year	2018	69	482	1		1	
	2019	133	739	1.257 (0.920, 1.719)	0.151	1.223 (0.889, 1.682)	0.216
	2020	87	410	1.482 (1.053, 2.087)	0.024^*∗*^	1.631 (1.144, 2.326)	0.007^*∗*^
	2021	166	709	1.636 (1.207, 2.216)	0.001^*∗*^	1.698 (1.242, 2.321)	0.001^*∗*^

Ward	Neonatal ward	134	438	2.096 (1.459, 3.010)	<0.001^*∗*^	1.076 (0.526, 2.206)	0.841
	Adult emergency	22	224	0.673 (0.394, 1.148)	0.146	0.765 (0.443, 1.321)	0.337
	Gyn and obstetrics	16	90	1.218 (0.659, 2.250)	0.529	1.807 (0.953, 3.425)	0.070
	Surgical and recovery	19	162	0.804 (0.457, 1.414)	0.448	1.365 (0.747, 2.494)	0.311
	Pediatric emergency	163	645	1.731 (1.219, 2.459)	0.002^*∗*^	1.219 (0.836, 1.778)	0.303
	Pediatric ward	54	459	0.806 (0.532, 1.222)	0.310	0.886 (0.577, 1.360)	0.580
	Adult medical ward	47	322	1		1	

*Note*. AOR: adjusted odds ratio; CI: confidence interval; COR: crude odds ratio; OR: odds ratio; Gyn: gynecology. ^*∗*^Significant association.

**Table 3:** Distribution of bacteria recovered from blood samples with age category of bacteremia-suspected patients at the UoG-CSH.

Bacterial isolates		Age category						
		<30 days (*n* = 580)	1 MTH–<1 yr. (*n* = 535)	1–<5 yrs. (*n* = 372)	5–12 yrs. (*n* = 281)	13–18 yrs. (*n* = 150)	19–45 yrs. (*n* = 635)	>45 yrs. (*n* = 242)
GPB (238)	*S. aureus* (*n* = 112)	18	35	22	10	4	15	8
	*Enterococcus* spp. (*n* = 49)	14	16	8	7	1	3	0
	CoNS (*n* = 23)	7	3	7	2	2	2	0
	*S. viridans* (*n* = 15)	1	4	5	2	1	2	0
	*S. pneumoniae* (*n* = 9)	2	5	2	0	0	0	0

Prevalence of GPB No (%)		42 (7.24)	63 (11.78)	44 (11.83)	21 (7.47)	8 (5.33)	22 (3.46)	8 (3.3)

GNB (247)	*K. pneumoniae* (*n* = 119)	56	23	17	9	3	7	4
	*E. coli* (*n* = 44)	11	8	8	2	0	12	3
	*Acinetobacter* spp. (*n* = 30)	13	3	9	2	0	3	0
	*Pseudomonas* spp. (*n* = 20)	6	9	0	1	0	4	0
	*Citrobacter* spp. (*n* = 13)	4	3	4	1	1	0	0
	Other NLFs (*n* = 21)	5	5	1	;22	2	3	3

Prevalence of GNB No (%)		95 (16.38)	51 (9.53)	39 (10.5)	17 (6.05)	6 (4)	29 (4.57)	10 (4.13)

Overall prevalence No (%)		137 (23.62)	114 (21.3)	83 (22.3)	38 (13.5)	14 (9.33)	51 (8.03)	18 (7.44)

*Note*. GNB: Gram-negative bacteria; GPB: Gram-positive bacteria; MTH: months; yrs.: years; spp.: species; NLFs: nonlactose fermenters; CoNS: coagulase-negative *Staphylococcus*.

**Table 4:** Antimicrobial susceptibility pattern of GPB isolated from bacteremia-suspected patients at the UoG-CSH.

Antibiotics	*S. aureus*		*Enterococcus* species		CoNS		*S. viridans*		*S. pneumoniae*		Total (%)	
	S	R	S	R	S	R	S	R	S	R	S (%)	R (%)
PEN	9	76	5	19	3	10	0	1	1	4	18 (14.1)	110 (85.9)
AMP/AMX	0	1	1	3	2	0	1	4	—	—	4 (33.3)	8 (66.7)
PIP/TAZ	0	1	1	4	—	—	—	—	—	—	1 (26.7)	5 (83.3)
CRX	9	17	8	9	2	5	4	4	2	3	25 (39.7)	38 (60.3)
CRO	2	2	0	3	3	0	1	1	—	—	6 (50.0)	6 (50.0)
OXA	1	0	0	1	0	1	0	1	2	1	3 (42.9)	4 (57.1)
CTX	32	28	—	—	4	5	1	1	—	—	37 (52.1)	34 (47.9)
CIP	22	6	2	1	3	4	1	1	0	1	28 (68.3)	13 (31.7)
MER	5	5	1	4	0	1	0	2	1	1	7 (35.0)	13 (65.0)
SXT	—	—	7	1	1	0	—	—	0	1	8 (80.0)	2 (20.0)
GEN	11	11	1	6	1	4	2	1	1	1	16 (41.0)	23 (59.0)
AMK	39	19	2	2	8	1	1	2	—	—	50 (67.6)	24 (32.4)
CLI	8	7	1	0	—	—	2	1	3	0	14 (63.6)	8 (36.4)
CAF	16	1	13	2	6	3	4	2	4	0	43 (84.3)	8 (15.7)
DOX	9	0	22	8	5	1	2	2	3	0	41 (78.8)	11 (11.2)
ERY	3	3	—	—	—	—	0	2	0	1	3 (33.3)	6 (66.7)
TOB	8	6	0	2	1	3	—	—	—	—	9 (45)	11 (55)
TET	5	18	5	13	0	5	2	5	2	3	14 (24.1)	44 (75.9)
Total (%)	179 (41.1)	201 (52.9)	69 (46.9)	78 (53.1)	39 (47.6)	43 (52.4)	21 (41.2)	30 (58.8)	19 (54.3)	16 (45.7)	327 (47.05)	368 (52.95)

*Note*. PEN: penicillin; AMP/AMX: ampicillin/amoxicillin; PIP/TAZ: piperacillin/tazobactam; CRX: cefuroxime; CRO: ceftriaxone; OXA: oxacillin; CTX: cefoxitin; CIP: ciprofloxacin; MER: meropenem; SXT: trimethoprim-sulfamethoxazole; GEN: gentamicin; AMK: amikacin; CLI: clindamycin; CAF: chloramphenicol; DOX: doxycycline; ERY: erythromycin; TOB: tobramycin; TET: tetracycline; S: sensitive; R: resistant; CoNS: coagulase-negative *Staphylococcus aureus*.

**Table 5:** Antimicrobial susceptibility pattern of GNB isolated from bacteremia-suspected patients at the UoG-CSH.

Antibiotics	*K. pneumoniae*		*E. coli*		*Acinetobacter*		*Pseudomonas* species		*Citrobacter* species		Other NLF rods		Total (%)	
	S	R	S	R	S	R	S	R	S	R	S	R	S	R
AMP/AMX	7	13	2	5	0	2	0	5	0	2	0	13	9 (18.4)	40 (81.6)
PIP/TAZ	6	50	1	13	4	7	3	2	5	4	0	6	19 (18.8)	82 (81.2)
CAZ	15	26	11	0	2	6	9	1	2	3	1	2	40 (51.3)	38 (48.7)
CRX	8	35	5	13	2	11	3	8	3	4	3	8	24 (23.3)	79 (76.7)
CRO	5	46	2	20	2	16	4	6	2	5	2	0	17 (15.5)	93 (84.5)
OXA	5	16	1	14	0	12	1	5	2	3	2	9	11 (15.7)	59 (84.3)
CIP	2	1	—	—	—	—	0	1	—	—	0	1	2 (40.0)	3 (60.0)
MER	24	51	11	16	11	10	8	3	6	5	8	4	68 (43.3)	89 (56.7)
SXT	33	16	15	1	5	3	6	0	8	0	3	3	70 (75.3)	23 (24.7)
GEN	11	74	11	21	4	8	6	4	2	7	3	9	37 (23.1)	123 (76.9)
AMK	29	69	19	21	10	20	13	4	1	6	6	10	78 (37.5)	130 (62.5)
CLI	21	14	9	6	11	3	4	0	1	0	1	2	47 (65.3)	25 (34.7)
CAF	5	2	—	—	—	—	—	—	—	—	2	1	7 (70.0)	3 (30.0)
DOX	7	11	11	1	1	5	0	1	0	1	2	1	21 (51.2)	20 (48.8)
ERY	2	3	0	1	—	—	—	—	—	—	—	—	2 (33.3)	4 (66.7)
TET	12	29	4	9	0	4	1	2	2	6	0	3	19 (26.4)	53 (73.6)
Total (%)	192 (29.6)	456 (70.4)	102 (19)	141 (20)	52 (32.7)	107 (67.3)	58 (20)	42 (19)	34 (42.5)	46 (57.5)	33 (31.4)	72 (68.6)	471 (36.4)	824 (63.6)

*Note*. AMP/AMX: ampicillin/amoxicillin; PIP/TAZ: piperacillin/tazobactam; CAZ: ceftazidime; CRX: cefuroxime; CRO: ceftriaxone; OXA: oxacillin; CIP: ciprofloxacin; MER: meropenem; SXT: trimethoprim-sulfamethoxazole; GEN: gentamicin; AMK: amikacin; CLI: clindamycin; CAF: chloramphenicol; DOX: doxycycline; ERY: erythromycin; TET: tetracycline; S: sensitive; R: resistant; NLF: nonlactose fermenter.

**Table 6:** Multidrug-resistance profile of bacterial isolates from blood culture of bacteremia-suspected patients at the UoG-CSH.

Bacterial isolates		Antibiotic resistance (%)						MDR = ≥R3 (%)
		R0	R1	R2	R3	R4	≥R5	
GPB	*Staphylococcus aureus* (*n* = 112)	15	40	29	16	11	1	28 (25.0)
	*Enterococcus* species (*n* = 49)	5	15	14	13	2	0	15 (30.6)
	CoNS (*n* = 23)	4	8	5	3	1	2	6 (26.1)
	*Streptococcus viridans* (*n* = 15)	2	2	5	2	3	1	6 (40.0)
	*Streptococcus pneumoniae* (*n* = 9)	2	2	2	2	0	1	3 (33.3)
	Total (*n* = 208)	28 (13.5)	67 (32.2)	55 (26.4)	36 (17.3)	17 (8.2)	5 (2.4)	58 (27.9)

GNB	*Klebsiella pneumoniae* (*n* = 119)	6	11	24	32	27	19	78 (65.5)
	*Escherichia coli* (*n* = 44)	6	5	10	9	7	7	23 (52.3)
	*Acinetobacter* species (*n* = 30)	2	4	7	7	8	2	17 (56.7)
	*Pseudomonas* species (*n* = 20)	4	6	6	1	2	1	4 (20.0)
	*Citrobacter* species (*n* = 13)	2	2	2	1	2	4	7 (53.8)
	Other NLFs (*n* = 21)	0	2	8	6	3	2	11 (52.4)
	Total (*n* = 247)	20 (8.1)	30 (12.1)	57 (23.1)	56 (22.7)	49 (19.8)	35 (14.2)	140 (56.7)

Grand total (*n* = 455)		48 (10.5)	97 (21.3)	112 (24.6)	92 (20.2)	66 (14.5)	40 (8.8)	198 (43.5)

*Note*. GNB: Gram-negative bacteria; GPB: Gram-positive bacteria; NLFs: nonlactose fermenters; CoNS: coagulase-negative *Staphylococcus*; R0: susceptible to all antibiotics; R1, R2, R3, and R4: resistant to 1, 2, 3, and 4 classes of antibiotics, respectively; ≥R5: resistant to 5 or more classes of antibiotics; ≥R3: resistance to 3 or more classes of antibiotics; MDR: multidrug resistant.

## Data Availability

All data generated or analyzed during this study were included in this article.

## Abbreviations

AMR: Antimicrobial resistance
AST: Antimicrobial susceptibility testing
BSI: Bloodstream infection
CI: Confidence interval
CLSI: Clinical Laboratory Standards Institute
GNB: Gram-negative bacteria
GPB: Gram-positive bacteria
LMICs: Low- and middle-income countries
MDR: Multidrug resistance
SD: Standard deviation
SPSS: Statistical Package for Social Sciences
SSA: Sub-Saharan Africa
UoG-CSH: University of Gondar Comprehensive Specialized Hospital
WHO: World Health Organization.
